# Preclinical diagnosis of Alzheimer’s disease: Prevention or prediction?

**DOI:** 10.1590/S1980-57642010DN40400002

**Published:** 2010

**Authors:** Ricardo Nitrini

**Affiliations:** MD, PhD, Behavioral and Cognitive Neurology Unit, Department of Neurology, and Cognitive Disorders Reference Center (CEREDIC). Hospital das Clínicas of the University of São Paulo School of Medicine, São Paulo SP, Brazil.

**Keywords:** Alzheimer’s disease, preclinical diagnosis, biomarkers

## Abstract

The diagnosis of Alzheimer’s disease (AD) for cases with dementia may be too late to allow effective treatment. Criteria for diagnosis of preclinical AD suggested by the Alzheimer’s Association include the use of molecular and structural biomarkers. Preclinical diagnosis will enable testing of new drugs and forms of treatment toward achieving successful preventive treatment. But what are the advantages for the individual? To know that someone who is cognitively normal is probably going to develop AD’s dementia when there is no effective preventive treatment is definitely not good news. A research method whereby volunteers are assigned to receive treatment or placebo without knowing whether they are in the control or at-risk arm of a trial would overcome this potential problem. If these new criteria are used wisely they may represent a relevant milestone in the search for a definitive treatment for AD.

## Abstract (pt)

O diagnóstico da doença de Alzheimer (DA) quando já existe demência pode ser tarde demais para permitir um tratamento eficaz. Critérios para o diagnóstico pré-clínico da AD sugeridos pela Associação de Alzheimer incluem o uso de biomarcadores moleculares e estruturais. O diagnóstico pré-clínico possibilitará avaliar novos medicamentos e formas de tratamento para alcançar o sucesso do tratamento preventivo. Mas quais são as vantagens do diagnóstico pré-clínico para o indivíduo? Receber a informação de que provavelmente irá se manifestar a demência da DA quando não existe um método eficaz de prevenção não é definitivamente uma boa notícia. Um método de pesquisa onde os voluntários sejam incluídos para receber tratamento ou placebo, sem saber se estão no grupo controle ou no grupo de risco, pode ser uma maneira de resolver este problema. Se esses novos critérios forem utilizados com sabedoria serão um marco relevante na busca do tratamento definitivo da DA.

Considerable progress has been made in understanding the pathophysiology of Alzheimer’s disease (AD) in the last 40 years, but effective treatments are not yet available.

The current diagnosis of AD is based on the criteria of the National Institute of Neurological and Communicative Diseases and Stroke-Alzheimer Disease and Related Disorder Association, which were published by McKhann and colleagues in 1984,^1^ or on the criteria of the Diagnostic and Statistical Manual (DSM) of Mental Disorders of the American Psychiatric Association (1994).^2^ According to these criteria, the diagnosis of Alzheimer’s disease should be made only when dementia is present, which is characterized by decline of memory and of at least one other cognitive domain. To be characterized as dementia, this cognitive decline must be sufficiently severe to interfere with social or professional activities of the individual.^1,2^

The diagnosis of AD when there is dementia (or major neurocognitive disorder, a new designation for dementia that may be included in the DSM-V)^3^ may be too late to allow effective treatment of the disease. The 1990s saw a growing interest in mild cognitive impairment (MCI), a condition where there is high risk for developing the dementia of AD.^4^ One of the main reasons for this interest was that it could represent a time window when preventive therapies could be more effective to avoid or delay the emergence of dementia. However, even MCI may represent a relatively advanced stage of disease in the central nervous system, as is revealed by structural neuroimaging using magnetic resonance imaging (MRI) and by the failure of clinical trials designed to postpone the conversion of MCI to the dementia of AD.^5^ Suggestions of new criteria for the diagnosis of AD have been proposed, incorporating biomarkers and neuroimaging findings.^6^

## Preclinical AD

Some evidence indicates the neuropathological changes of AD begin years before the appearance of any neuropsychiatric symptoms. Cognitively normal elderly individuals, without complaints or objective evidence of cognitive decline, may have neuropathological changes in the brain consistent with the diagnosis of AD, indicating that there is a preclinical stage of the disease.^7^

Criteria for diagnosis of preclinical AD were suggested by a workgroup sponsored by the Alzheimer’s Association and recently presented at the International Conference on AD and Related Disorders (ICAD) in Hawaii. A preliminary version was then submitted to the community for appraisal.^8^

These criteria hold that preclinical diagnosis of AD is possible with the use of biomarkers, which are able to, directly or indirectly, identify the presence of neuropathological changes of AD.^8^

## Biomarkers

The main pathophysiological hypothesis for AD assumes that Aβ_42_ initiates a process called the amyloid cascade.^9^ Although the exact mechanisms have yet to be elucidated, the toxic effects of oligomeric forms of this peptide and its deposition as protofibrils and fibrils in the brain to form the amyloid plaques is followed by synaptic dysfunction and the appearance of intracellular neurofibrillary tangles containing hyperphosphorylated tau protein. Two types of biomarkers can be identified at this stage when there are still no clinical or macroscopic changes of the disease. The first evaluates the concentrations of Aβ_42_ and tau protein (or phospho-tau) in cerebrospinal fluid (CSF). Low CSF-concentration of Aβ_42_, associated with high CSF-concentration of tau protein, has high sensitivity and specificity for the diagnosis of AD. The second method is based on a radioactive tracer injected into the blood stream that crosses the blood brain barrier and binds to fibrillar forms of Aβ_42_. The amount and location of this radioactive tracer can be assessed with positron emission tomography (PET). These two biomarkers have been called *molecular* biomarkers of AD. With disease progression, characteristic hypometabolism in the temporal-parietal pattern may be seen with fluoro-deoxy-glucose (FDG) PET, and later, brain atrophy can be identified with MRI. At this stage of preclinical AD, cognition may be entirely normal or only very subtle signs of cognitive decline may be present.^8^

## Advantages of preclinical diagnosis

Although these markers are not ideal, albeit due to their complexity or cost, or to the fact that their sensitivity and specificity for predicting the actual risk of developing MCI or AD are not yet well established, they may be extremely useful for identifying asymptomatic individuals at high risk of AD and for allowing the search for new drugs or other methods such as vaccines or genetic manipulations that might slow or prevent progress of AD.

There are numerous examples in Medicine where advances in methods for early diagnosis have changed the evolution of diseases. The first examples that spring to mind are the early diagnosis of cancer or the treatment of hypertension or hyperlipidemia for preventing myocardial infarction or stroke. Yet there is an example which more closely resembles AD because it is also related to a cause of dementia.

## A historical perspective

Up until the mid-20^th^ century, neurosyphilis was the most frequent cause of dementia. The discovery of the etiologic agent of syphilis in 1905 and of the complement fixation reaction for diagnosis of syphilis in 1906 were very important for the extraordinary reduction of the frequency of neurosyphilis that occurred after only a few short decades.^10,11^

The complement fixation reaction for the diagnosis of syphilis or Wassermann’s reaction was the first biomarker that allowed evaluation of the results of different types of treatments. From the arsenicals introduced by Paul Ehrlich, to the malaria therapy described by Julius Wagner-Jauregg, and finally the penicillin discovered by Alexander Fleming, to name only three Nobel Laureates, the existence of biomarkers was key to following up the efficacy of the treatments.^11^ For neurosyphilis, besides the Wassermann’s reaction in blood and CSF, there were also other biomarkers in CSF allowing diagnosis of asymptomatic neurosyphilis and monitoring of the outcome of treatments that were able to prevent the onset of dementia and other neurological disorders.^12^ It took less than 40 years from the discovery of the first biomarker, the Wassermann’s reaction, to achieve the successful treatment of neurosyphilis.

It should be noted that to be truly effective, the treatment of neurosyphilis needs to be carried out in the asymptomatic phase, as treatment in later stages when dementia is already present can stabilize the clinical picture, and usually precludes full recovery from the cognitive and behavioral abnormalities.^12^ This observation reinforces the notion that for AD also, treatment probably has to be carried out before the appearance of the clinical manifestations.

These historical facts strengthen the value of the preclinical diagnosis of AD. It is highly probable that with biomarkers it will be possible to test new drugs and new forms of treatments to achieve successful preventive treatment in the years to come. Aging without AD will be one of the most important triumphs of the medical sciences, and will probably cause deep changes in human society.

## The individual at risk

However, there is an aspect that deserves particular concern on this point: What about the advantages and disadvantages of the preclinical diagnosis for the individual? To know that someone without cognitive impairment is going to develop the dementia of AD when there is no current available treatment is definitely not good news. We are faced with a dilemma: to test new treatments for this condition and thereby reach a cure or delay the onset of dementia is very important for humankind. However, being aware of one’s preclinical diagnosis of AD is not very useful for an individual who is cognitively normal, and may even have a destabilizing effect. Clearly, biomarkers are undoubtedly relevant for confirming diagnosis in cases where cognitive decline is already present.

Borrowing from history again, it is well known that there are conditions where the preclinical diagnosis is possible, but does not represent any clear advantage. To remain in the field of dementia, the best example is perhaps Huntington’s disease. In this autosomal dominant disease, it is possible to ascertain whether the individual has inherited the mutation by doing a genetic test. The accuracy of the genetic test is almost 100% in predicting if the individual is going to develop the clinical disease or not. As an effective treatment is not yet available, the decision to be submitted to the genetic test is very fraught with problems. Accordingly, the Huntington’s Disease Society of America states, “the decision whether to test or not is intensely personal and there is no ‘right’ answer.” ^13^

The situation is even more difficult in AD, because the accuracy of the preclinical diagnosis is not known and is lower than in Huntington’s disease. It should be pointed out that in the preliminary version of the Criteria for Preclinical Diagnosis of AD suggested by the Alzheimer’s Association it is clearly stated that “these are not intended to serve as diagnostic criteria for clinical purposes”.^8^

## A possible approach

To be able to test for new treatments of individuals at risk without unnecessarily discovering an unexpected risk for which there is no available treatment may be possible by using a research method where volunteers in their fifties or sixties are included to receive treatment or placebo without knowing whether they are in the control or at-risk arm of the trial. Naturally, any individual is entitled to be informed of their risk status, but only after the limitations of the diagnostic tests as well as the absence of effective treatment, have been made clear.

If these new criteria are adopted wisely they may represent a relevant milestone in the search for the definitive treatment of AD.
